# Potential of ^99m^Tc-MIBI SPECT imaging for evaluating non-alcoholic steatohepatitis induced by methionine-choline-deficient diet in mice

**DOI:** 10.1186/s13550-014-0057-z

**Published:** 2014-10-09

**Authors:** Takemi Rokugawa, Tomoya Uehara, Yusuke Higaki, Shuuichi Matsushima, Atsushi Obata, Yasushi Arano, Kohji Abe

**Affiliations:** Department of Drug Metabolism and Pharmacokinetics, Research Laboratory for Development, Shionogi & Co., Ltd., 3-1-1, Futaba-cho, Toyonaka, Osaka 561-0825 Japan; Department of Molecular Imaging and Radiotherapy, Graduate School of Pharmaceutical Sciences, Chiba University, 1-8-1 Inohana, Chuo-ku, Chiba, 260-8675 Japan; Department of Pharmaceutical Analytical Chemistry, Graduate School of Medicine, Dentistry, and Pharmaceutical Sciences, Okayama University, Okayama, 1-1-1, Tsushima naka, Kita-ku, Okayama 700-8530 Japan; Department of Drug Safety Evaluation, Research Laboratory for Development, Shionogi & Co., Ltd., 3-1-1, Futaba-cho, Toyonaka, Osaka 561-0825 Japan

**Keywords:** ^99m^Tc-MIBI, NASH, Mice, MCD diet

## Abstract

**Background:**

Hepatic mitochondrial dysfunction has been implicated in pathological conditions leading to non-alcoholic steatohepatitis (NASH). Technetium-99 m-2-methoxyisobutyl-isonitrile (^99m^Tc-MIBI), a lipophilic cationic myocardial perfusion agent, is retained in the mitochondria depending on membrane potential. The aim of this study was to investigate the feasibility of ^99m^Tc-MIBI for evaluating the hepatic mitochondrial dysfunction induced by methionine-choline-deficient (MCD) diet in mice.

**Methods:**

Male C57Black6J/jcl mice were fed a MCD diet for up to 4 weeks. SPECT scan (*N* =6) with ^99m^Tc-MIBI was performed at 2 and 4 weeks after MCD diet. Mice were imaged with small-animal SPECT/CT under isoflurane anesthesia. Radioactivity concentrations of the liver were measured, and the time of maximum (*T*_max_) and the elimination half-life (*T*_1/2_) were evaluated. After SPECT scan, liver histopathology was analyzed to evaluate steatosis and inflammation. Non-alcoholic fatty liver disease (NAFLD) activity score was obtained from the histological score of hepatic steatosis and inflammation. Blood biochemistry and hepatic ATP content were also measured (*N* =5 to 6).

**Results:**

Plasma alanine aminotransferase (ALT) and aspartate aminotransferase (AST) levels were significantly elevated at 2 and 4 weeks after MCD diet. A decrease in hepatic ATP content was also observed in MCD-fed mice. ^99m^Tc-MIBI SPECT imaging clearly showed the decrease of hepatic ^99m^Tc-MIBI retention in MCD-fed mice compared to control mice. *T*_1/2_ after ^99m^Tc-MIBI injection was significantly decreased in the liver of MCD-fed mice (control, MCD 2 weeks, and MCD 4 weeks, *T*_1/2_ = 57.6, 37.6, and 19.8 min, respectively), although no change in *T*_max_ was observed in MCD-fed mice. SPECT data and histological score showed that the negative correlation (*r* = −0.74, *p* <0.05) between *T*_1/2_ and NAFLD activity score was significant.

**Conclusions:**

Hepatic ^99m^Tc-MIBI elimination was increased with increase in NAFLD activity score (NAS) in mice fed MCD diet for 2 and 4 weeks. These results suggest that ^99m^Tc-MIBI SPECT imaging might be useful for detecting hepatic mitochondrial dysfunction induced by steatosis and inflammation such as NAFLD or NASH.

## Background

Non-alcoholic fatty liver disease (NAFLD) is one of the most common forms of chronic liver disease recognized as a hepatic manifestation of metabolic syndrome without a history of alcoholic abuse [1]. NAFLD encompasses a wide spectrum of conditions ranging from simple steatosis to non-alcoholic steatohepatitis (NASH) with or without fibrosis, cirrhosis, and hepatocellular carcinoma [2]. A two-hit theory has been proposed for NASH pathogenesis. The first hit refers to factors that promote hepatic steatosis, and the second hit refers to factors leading from hepatic steatosis to steatohepatitis [3,4]. One of the second hit factors is the formation of reactive oxygen species (ROS) [5,6]. ROS directly damages respiratory chain polypeptides and oxidizes the unsaturated lipid of cytoplasmic hepatic fat deposits to cause lipid peroxidation. Both ROS and lipid peroxidation products attack mitochondrial DNA [7]. Oxidative mitochondrial DNA lesions and mitochondrial DNA depletion may impair the synthesis of respiratory chain polypeptides. These effects may further block the flow of electrons in the respiratory chain to further increase mitochondrial ROS formation and decrease the mitochondrial membrane potential [5,8]. Thus, NASH is considered to be a mitochondrial disease because mitochondrial dysfunction in the liver would be involved in all successive steps in the induction of NASH [9]. It has been reported that patients with NASH present ultrastructural mitochondrial alterations [10], impairment of hepatic ATP synthesis [11], and increased ROS production [12,13]. Therefore, mitochondrial dysfunction is considered to have an important role in the liver during NASH progression since mitochondrial dysfunction causes overproduction of ROS inducing lipid peroxidation, inflammation, and cell death.

Technetium-99 m-2-methoxy-isobutyl-isonitrile (^99m^Tc-MIBI) is a SPECT imaging probe which is used for myocardial perfusion imaging [14]. This lipophilic cationic imaging probe predominantly accumulates in the mitochondria, where it is retained in response to the electrical potential generated across the membrane bilayer [15–17]. Therefore, ^99m^Tc-MIBI retention in the mitochondria might be related to mitochondrial function [15,18]. Masuda et al. have recently reported that ^99m^Tc-MIBI scintigraphy is useful for discriminating NASH from simple steatosis in clinical studies [19]. These findings suggest the possibility of ^99m^Tc-MIBI imaging for the evaluation of hepatic mitochondrial function in the liver disease state such as NASH and NAFLD. The present study was undertaken to investigate the feasibility of ^99m^Tc-MIBI imaging for evaluating hepatic dysfunction using mice fed methionine-choline-deficient (MCD) diet, one of the most commonly used NASH animal models [20,21].

## Methods

### Animals and MCD diet

Male C57Black6J/jcl mice, aged 8 weeks, were purchased from CLEA Japan (Shizuoka, Japan). Mice were studied at 2 and 4 weeks after MCD diet (Dyets, Bethlehem, PA, USA) feeding or normal diet feeding (control group). They were allowed free access to chow and tap water and housed in a temperature-controlled room maintained on a 12-h light/dark cycle with lights on at 8:00 am. The experimental protocols were reviewed and approved by the Institutional Animal Care and Use Committee of Shionogi Research Laboratories, Osaka University Graduate School of Medicine, and Animal Care Committee of Chiba University.

### SPECT/CT imaging

**Figure 1:**
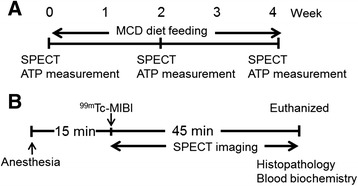
**Schematic diagram of the experimental protocol. (A)** MCD diet feeding. **(B)** SPECT imaging protocol.

SPECT imaging and X-ray CT imaging were performed with a small-animal SPECT/CT system (FX-3200, TriFoil Imaging Inc., Chatsworth, CA, USA) equipped with a five-pinhole (1.0 mm) collimator. ^99m^Tc-MIBI was prepared with a Cardiolite® kit (Fujifilm RI Pharma Co., Ltd., Tokyo, Japan) or purchased as Cardiolite® (Fujifilm RI Pharma Co., Ltd., Tokyo, Japan). Mice were anesthetized with 3% isoflurane and anesthesia was maintained with 1.5% isoflurane. Under isoflurane anesthesia, the venous catheter was introduced through the tail vein and used for the administration of ^99m^Tc-MIBI. As shown in Figure 1B, SPECT scans (*N* =6 per group) were started immediately after injection of ^99m^Tc-MIBI (30 ~ 60 MBq). Dynamic data acquisition was performed for 45 min by a two-scan sequence of 10 s per projection with stepwise rotation of two projections over 360°, followed by 150 s per projection with stepwise rotation of two projections over 360°.

All SPECT data were reconstructed by a 3D-ordered subset expectation maximization (3D-OSEM) algorithm method with two subsets and five iterations in FLEX-RECON software. Imaging data were analyzed using AMIDE 0.9.2 software. 3D region of interest (ROI) was put on the liver tissue except for the portal area and estimated liver time-activity curves (TACs). TACs were normalized to liver activity peak. The half-life (*T*_1/2_), peak time (*T*_max_), and area under the curve (AUC) for each individual animal were calculated from the data of TAC using WinNonlin. All the parameters were then averaged for all the control mice and the mice fed the MCD diet for 2 and 4 weeks. Because the hepatic TACs of ^99m^Tc-MIBI did not reach a constant level within 45 min, we used the first-order intermediate equation to fit the kinetic data and generate the best fitted liver curves [22].

### Hepatic ATP content

The content of hepatic ATP was measured by HPLC according to the method of Dai et al. [23]. Briefly, tissue samples were homogenized in homogenized buffer (0.3 M HClO_4_ and 1 mM EDTA-Na) and centrifuged at 14,000 rpm for 2 min. A 20-μL portion of the supernatant was injected into an HPLC system for ATP content determination. HPLC analysis was performed with Chemcosorb 5-I-C18 (4.6 mm × 300 mm, Chemco Scientific Co., Ltd., Osaka, Japan) at 1 mL/min. The composition of the mobile phase was 0.2 M NH_4_H_2_PO_4_ adjusted to pH =4.1 with 1 N HCl. The detection wavelength was 254 nm and the retention time of ATP was 16.3 min. ATP was calculated using an internal standard method.

### Histology and blood biochemistry

After SPECT scans, mice were sacrificed by exsanguination under isoflurane anesthesia. Plasma was collected and assayed for the content of alanine aminotransferase (ALT), aspartate aminotransferase (AST), triglyceride (TG), total cholesterol (TC), and high-density lipoprotein cholesterol (HDLC). The right hepatic lobes were fixed in 10% formalin and sectioned, and the sections were stained with hematoxylin and eosin (H&E). The liver histopathology was scored as follows: steatosis (0 to 4), inflammation (0 to 3), and ballooning (0 to 2). NAFLD activity score (NAS) was calculated by using the sum of each histological score.

### Statistics

Quantitative data were expressed as means ± standard error of the mean (SEM). Means were compared using Dunnett's test. *p* values <0.05 were considered statistically significant. The Pearson product-moment correlation coefficient was used to evaluate the relationship between *T*_1/2_ of ^99m^Tc-MIBI in the liver and the liver histological score or plasma.

## Results

### Physiological characteristics and hepatic pathology

**Figure 2:**
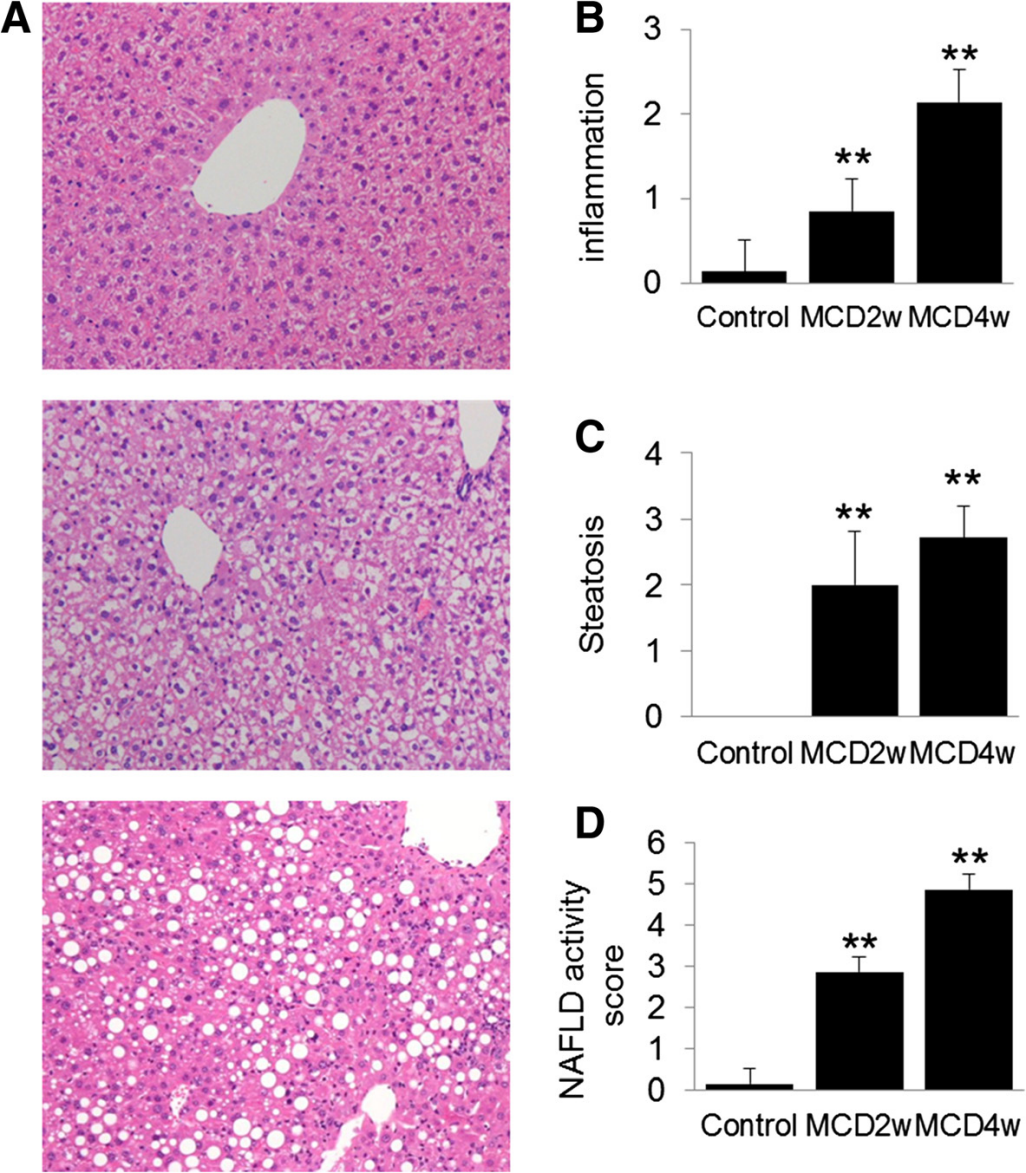
**Histochemical investigation from the liver of mice fed the normal or MCD diet for 2 or 4 weeks. (A)** Representative micrographs of hematoxylin and eosin (H&E). **(B)** Score of inflammation. **(C)** Score of steatosis. **(D)** NAFLD activity score. Data are expressed as mean ± SEM. Statistical difference was assessed using Dunnett's test. *p <0.05, **p <0.01 compared to the control mice.

Plasma ALT and AST levels were significantly elevated in mice fed MCD diet compared to control mice (*p* <0.05) (Table 1). Inflammation and steatosis were observed in mice fed MCD diet for 2 weeks. These pathological changes were aggravated by prolongation of the MCD diet (4 weeks) (Figure 2). Ballooning was not observed in MCD-fed mice.

**Table 1:** **Plasma parameters in mice fed control and MCD diet for 2 and 4 weeks**

**Plasma parameter**	**Control**	**MCD 2 weeks**	**MCD 4 weeks**
TC (mg/dL)	90.9 ± 2.55	57.7 ± 1.67**	32.9 ± 2.84**
TG (mg/dL)	110 ± 18.4	9.80 ± 1.77**	14.7 ± 1.81**
AST (IU/L)	63.8 ± 21.3	312 ± 84.5**	324 ± 65.8**
ALT (IU/L)	26.7 ± 8.02	191 ± 41.7**	239 ± 31.4**
HDLC (mg/dL)	50.9 ± 1.72	30.8 ± 1.58**	14.4 ± 1.63**

Data are expressed as mean ± SEM of five to seven experiments for each group. Statistical differences were assessed using Dunnett's test. TC, total cholesterol; TG, triglyceride; AST, aspartate transaminase; ALT, alanine aminotransferase; HDLC, high-density lipoprotein cholesterol. *p <0.05, **p <0.01 compared to the control mice.

### Hepatic ATP content

Hepatic ATP was significantly decreased in mice fed MCD diet for 2 weeks (2.57 ± 0.03 μmol/g tissue) and 4 weeks (2.47 ± 0.02 μmol/g tissue) compared with the control group (3.04 ± 0.15 μmol/g tissue) (Table 2).

**Table 2:** **Hepatic ATP content in mice fed control and MCD diet for 2 and 4 weeks**

**μmol/g tissue**	**Control**	**MCD 2 weeks**	**MCD 4 weeks**
ATP content	3.04 ± 0.15	2.57 ± 0.03*	2.47 ± 0.02**

Data are expressed as mean ± SEM. Statistical difference was assessed using Dunnett's test. *p <0.05, **p <0.01 compared to the control mice.

### SPECT imaging

**Figure 3:**
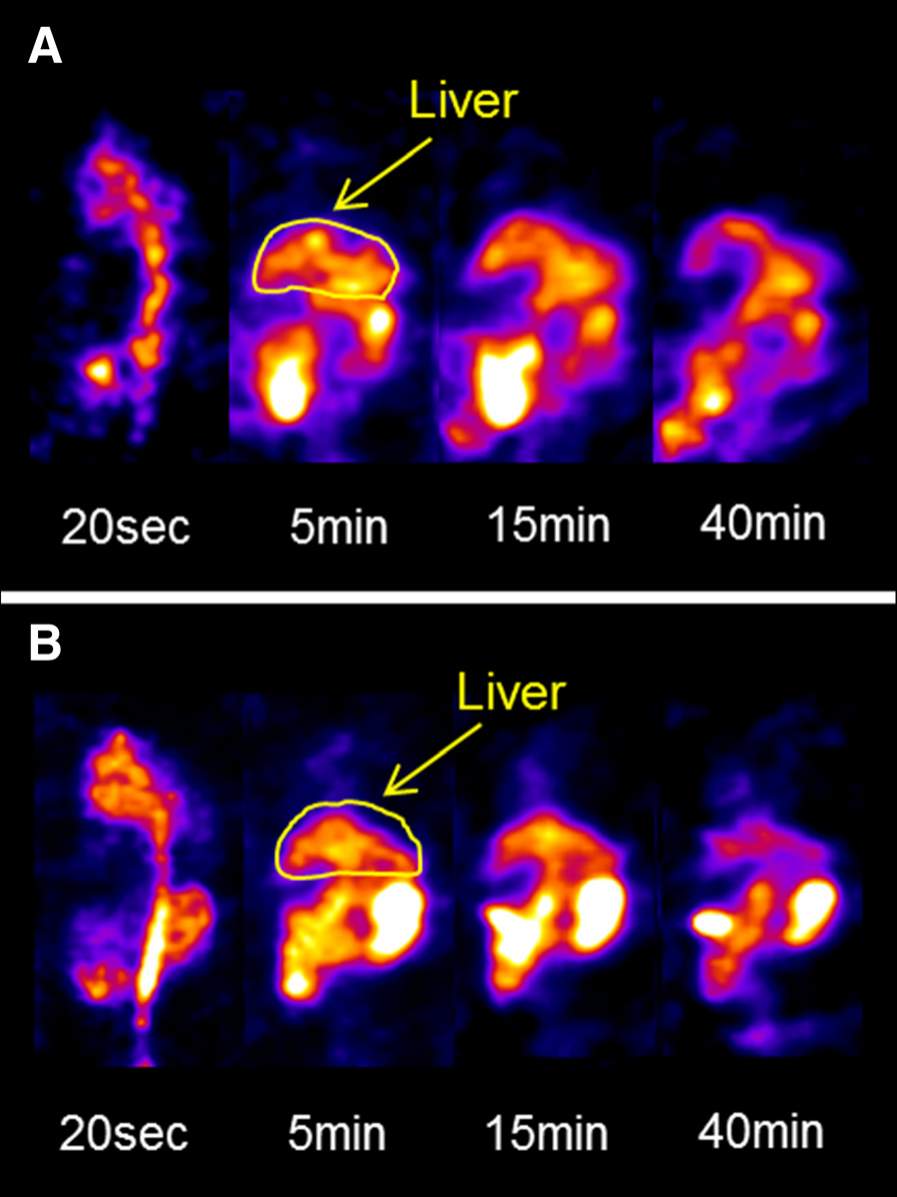
**Dynamic SPECT images after** ^**99m**^ **Tc-MIBI injection in control and MCD-fed mice. (A)** Mouse fed control diet. **(B)** Mouse fed MCD diet for 4 weeks.

**Figure 4:**
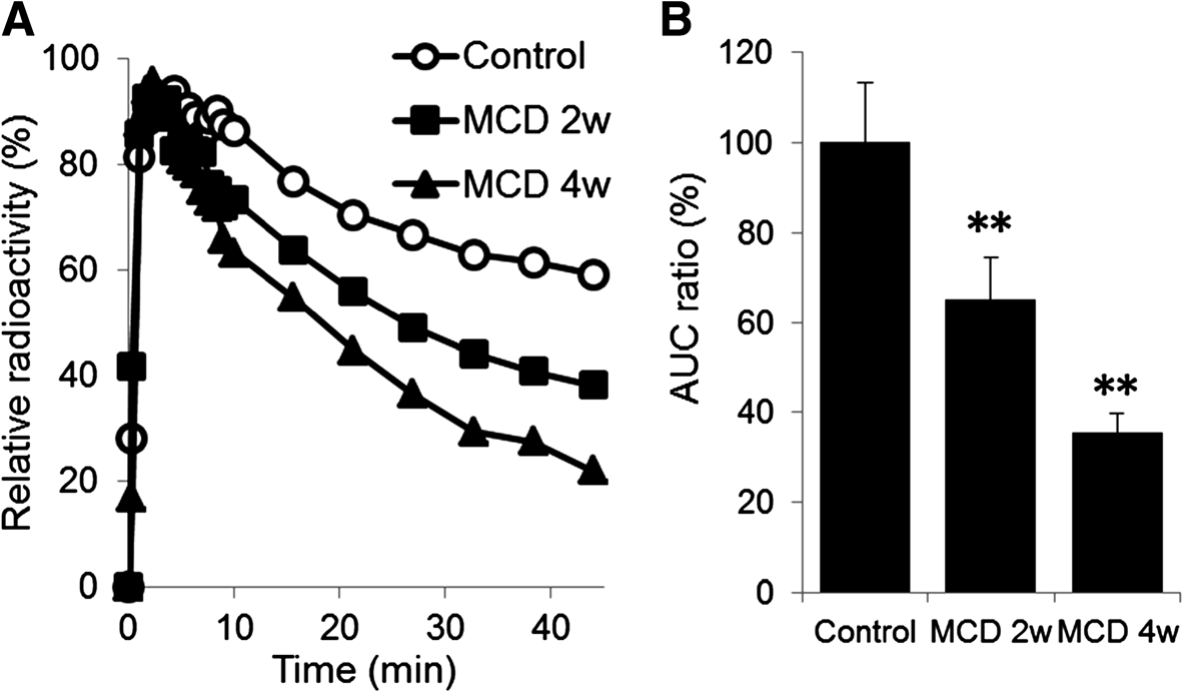
**Liver radioactivity after** ^**99m**^ **Tc-MIBI injection in mice fed the normal and MCD diet for 2 and 4 weeks.** (*N* =6 per group). **(A)** Time-activity curve of relative radioactivity. **(B)** AUC ratio compared to control. Radioactivity was normalized by liver peak activity. Data are expressed as mean ± SEM. Statistical difference was assessed using Dunnett's test. *p <0.05, **p <0.01 compared to the control mice.

**Figure 5:**
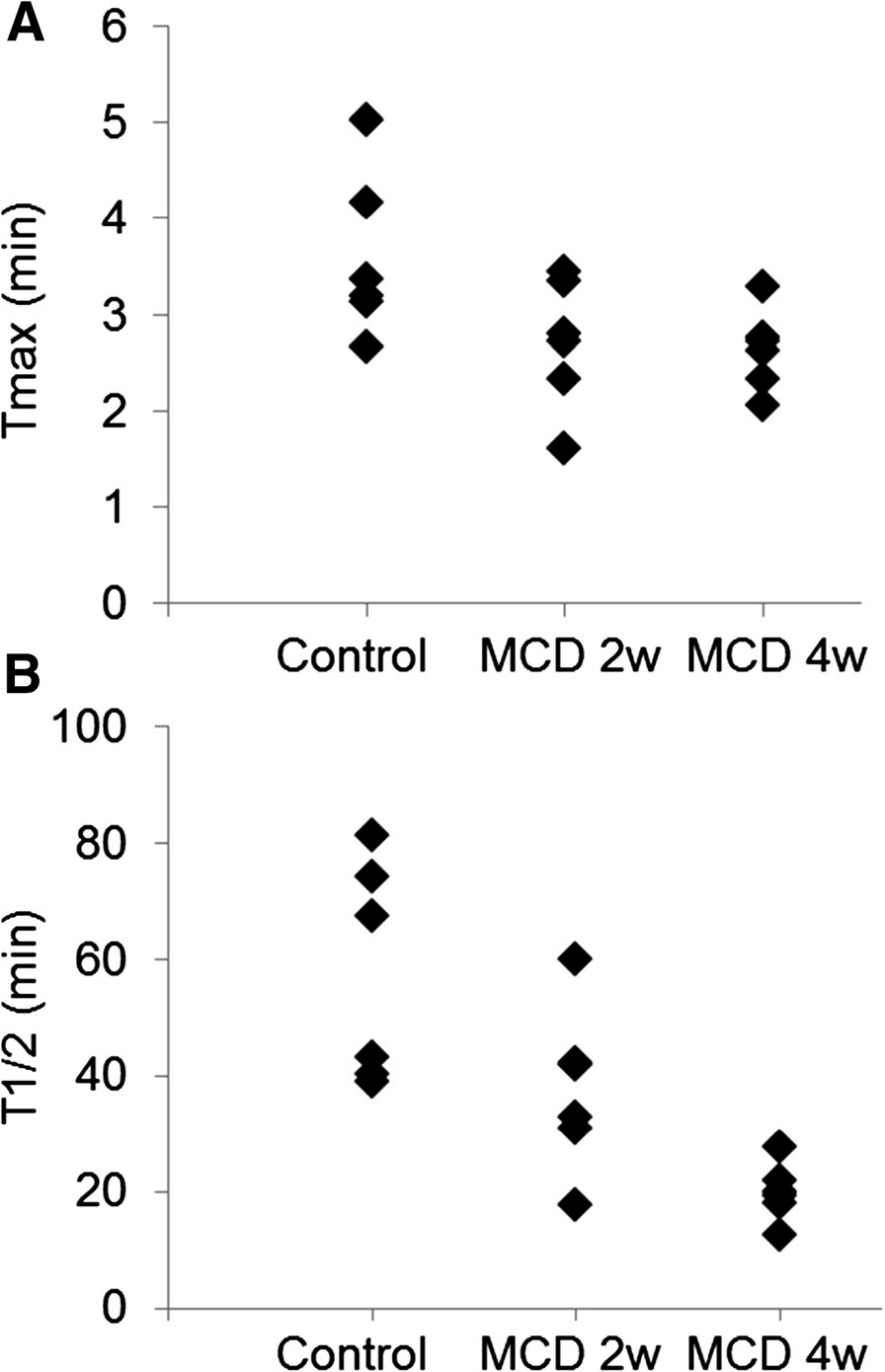
**Pharmacokinetic parameter after** ^**99m**^ **Tc-MIBI injection in mice fed control and MCD diet for 2 and 4 weeks. (A)** *T*_max_ in the liver after ^99m^Tc-MIBI injection. **(B)** *T*_1/2_ in the liver after ^99m^Tc-MIBI.

**Figure 6:**
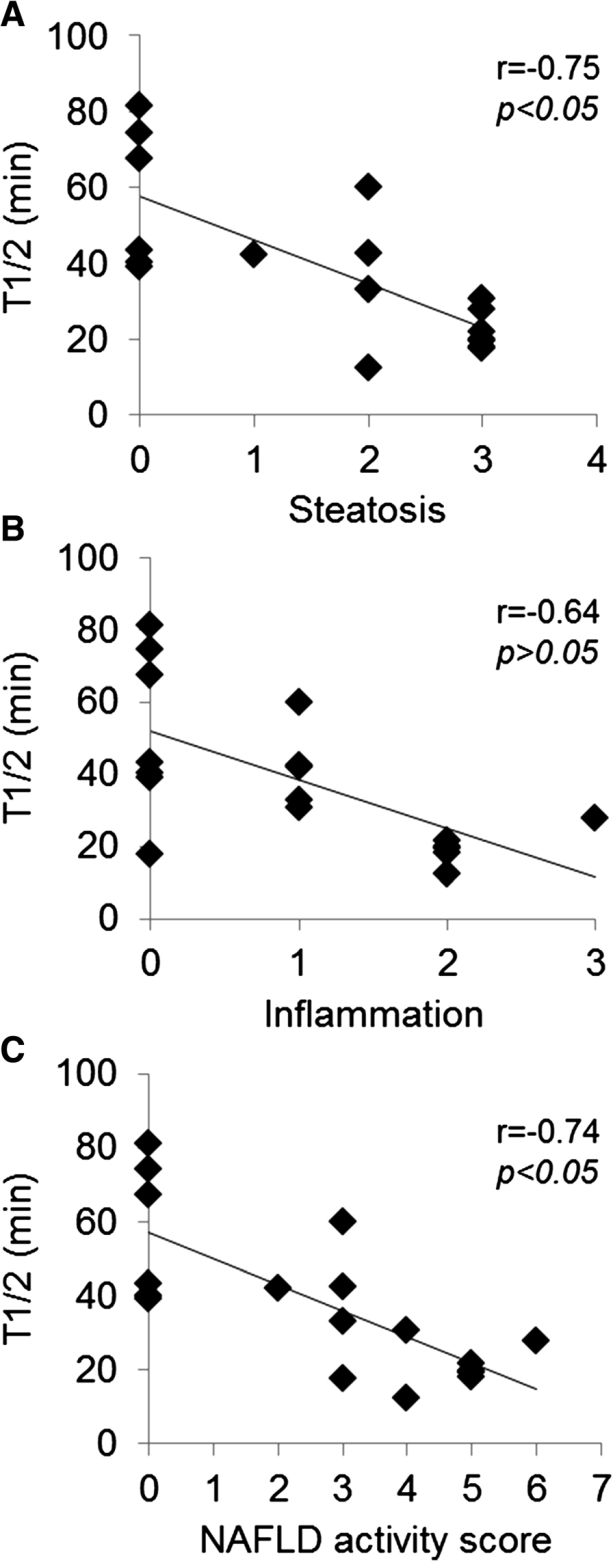
**Correlations between** ^**99m**^ **Tc-MIBI and histological data in mice fed control and MCD diet for 2 and 4 weeks. (A)** Correlation between ^99m^Tc-MIBI *T*_1/2_ and steatosis (*r* = −0.75, *p* <0.05). **(B)** Correlation between ^99m^Tc-MIBI *T*_1/2_ and inflammation (*r* = −0.64, *p* >0.05). **(C)** Correlation between ^99m^Tc-MIBI *T*_1/2_ and NAFLD activity score (*r* = −0.74, *p* <0.05). Correlation analysis was assessed using the Pearson product-moment correlation.

Coronal slice dynamic SPECT images and TACs of the liver are shown in Figures 3 and 4A. In the MCD-fed mice livers, the washout ratio was faster than that in the control mice. A significant decrease in the relative AUC was observed in mice fed MCD diet for 2 and 4 weeks (Figure 4B). As shown in Table 3 and Figure 5, *T*_1/2_ of ^99m^Tc-MIBI was significantly decreased in MCD diet mice compared with control mice. The value of *T*_1/2_ was 57.6 ± 7.73 in control mice, 37.6 ± 5.80 in mice fed MCD diet for 2 weeks (*p* <0.05), and 19.8 ± 2.05 in mice fed MCD diet for 4 weeks (*p* <0.01). However, no significant difference of *T*_max_ was observed between the control and MCD-fed groups. The correlation analysis between steatosis score and *T*_1/2_ indicated a negative correlation (*r* = −0.75, *p* <0.05). The correlation between NAFLD activity score and *T*_1/2_ was also negative (*r* = −0.74, *p* <0.05). The correlation between inflammation and *T*_1/2_ was mild (*r* = −0.64, *p* >0.05) (Figure 6). The correlation between *T*_1/2_ and AST or *T*_1/2_ and ALT was weak (*r* = −0.20 and −0.39, *p* >0.05, respectively).

**Table 3:** **Hepatic** ^**99m**^ **Tc-MIBI kinetics in mice fed control and MCD diet for 2 and 4 weeks**

**Parameter**	**Control**	**MCD 2 weeks**	**MCD 4 weeks**
*T*_1/2_ (min)	57.6 ± 7.73	37.6 ± 5.80*	19.8 ± 2.05**
*T*_max_ (min)	3.59 ± 0.35	2.71 ± 0.28	2.63 ± 0.17

Data are expressed as mean ± SEM. Statistical difference was assessed using Dunnett's test. *p <0.05, **p <0.01 compared to the control mice.

## Discussion

^99m^Tc-MIBI heart scintigraphy is used to evaluate cardiac mitochondrial dysfunction in patients with cardiomyopathy [24,25]. In addition, ^99m^Tc-MIBI leg scintigraphy has been used to detect mitochondrial dysfunction in progressive supranuclear palsy patient skeletal muscle [26]. ^99m^Tc-MIBI, a lipophilic cationic myocardial perfusion agent, is considered to be retained in the mitochondria by the higher membrane potential, and the loss of mitochondrial membrane potential results in rapid washout of ^99m^Tc-MIBI from the myocyte [14]. In the present study, we showed that rapid washout of ^99m^Tc-MIBI indicating mitochondrial dysfunction was observed in the liver of MCD-fed mice. The MCD-fed rodent model is widely used as a NASH model because of its similarity with human NASH pathology. Rodents fed MCD diet showed mitochondrial dysfunction such as reduction of the activity of mitochondrial respiratory chain and hepatic ATP depletion [27]. These findings suggest that the cause of rapid washout of ^99m^Tc-MIBI is the loss of mitochondrial membrane potential since ^99m^Tc-MIBI is retained to the mitochondrial membrane in response to membrane potential. Rogers et al. showed that prolonged exposure of preadipocytes to fatty acid led to mitochondrial dysfunction such as decrease of ATP content and reduction of mitochondrial inner membrane potential [28]. We also confirmed the decrease of ATP content in the liver of MCD-fed mice. Therefore, our and previous findings indicate that the rapid clearance of hepatic MIBI might be due to mitochondrial dysfunction including reduction of ATP content in MCD-fed mice. Regarding magnetic resonance imaging (MRI), it has been reported that *T*_max_ and *T*_1/2_ after injection of gadolinium-ethoxybenzyl-diethylenetriamine penta-acetic acid (Gd-EOB-DTPA) were significantly prolonged in a NASH rat model [29]. Furthermore, *T*_max_ and *T*_1/2_ after Gd-EOB-DTPA injection significantly correlated with the fibrosis rate [30]. It is well known that organic anion-transporting polypeptide (oatp) 1 mediates the uptake of Gd-EOB-DTPA and multidrug resistance-associated protein 2 (mrp2) mediates biliary excretion of Gd-EOB-DTPA in rats [31,32]. These reports suggest that the elimination of ^99m^Tc-MIBI also might be influenced by transporter expression in NASH pathology since ^99m^Tc-MIBI passively diffuses into hepatocytes and the biliary excretion is mediated by P-glycoprotein (P-gp) [33]. However, Canet et al. recently reported that the liver protein expression of P-gp did not alter in a MCD-fed mouse [34]. This report suggests that the role of efflux transporter via P-gp is considered to be small for ^99m^Tc-MIBI washout in this MCD-fed mouse.

In the present study, the most important finding is to show the negative correlation between ^99m^Tc-MIBI clearance (*T*_1/2_) and NAFLD activity score including steatosis and inflammation score. Steatosis and inflammation were histologically observed at 2 and 4 weeks after MCD diet in this study. The MCD diet also seemed to aggravate the inflammation at 4 weeks compared with that at 2 weeks after feeding, although the severity of steatosis was not different between 2 and 4 weeks. These data suggest that the ^99m^Tc-MIBI washout is likely to correlate with pathology including both steatosis and inflammation in MCD mice. Masuda et al. [19] have reported that hepatic ^99m^Tc-MIBI uptake is correlated with the NAFLD activity score in a clinical study. These non-clinical and clinical findings suggest that hepatic ^99m^Tc-MIBI SPECT imaging might be useful for evaluating NASH progression pathology such as mitochondrial dysfunction. However, further study will be needed to clarify the relation between mitochondrial membrane potential and ^99m^Tc-MIBI binding activity.

## Conclusions

Hepatic retention of ^99m^Tc-MIBI was decreased with increase in NAFLD activity score in MCD-fed mice. This study indicates that ^99m^Tc-MIBI SPECT imaging might be useful for evaluating hepatic mitochondrial dysfunction such as NAFLD or NASH.
